# Progress and prospects for herpesvirus vaccination using gB antigens

**DOI:** 10.3389/fimmu.2026.1827628

**Published:** 2026-05-29

**Authors:** Solomon English, Adam Khan-Qureshi, Alexander D. Douglas

**Affiliations:** 1 Jenner Institute, Nuffield Department of Medicine, University of Oxford, Oxford, United Kingdom; 2 Division of Structural Biology, Nuffield Department of Medicine, University of Oxford, Oxford, United Kingdom

**Keywords:** EBV, gB, HCMV, herpesvirus, HSV1, HSV2, vaccine, VZV

## Abstract

The success of vaccines against varicella zoster virus (VZV) demonstrates the feasibility of high-level efficacy against clinical consequences of herpesvirus infection, but there is a need for new vaccines against others – in particular Epstein-Barr Virus (EBV), human cytomegalovirus (HCMV) and herpes simplex virus 1 and 2 (HSV). Herpesviruses use surface glycoproteins to trigger membrane fusion during cell invasion. Glycoprotein B (gB) is conserved across the family and acts as the fusogen. Vaccines based upon viral fusion proteins protect against many other viruses, and a gB-targeting HCMV vaccine achieved partial efficacy in clinical trials. This experience encourages development of improved gB-based antigens and formulations. There has recently been progress in stabilisation of the pre-fusion conformation of gB, which may be the more relevant structure with respect to immunological protection. Nonetheless gB-targeting vaccines have received less attention recently than vaccines against receptor-binding glycoproteins such as EBV gH/gL and gp350, and HCMV pentamer. We therefore seek to review current knowledge regarding gB as a vaccine antigen: understanding of herpesvirus gB structure and function, within the context of the wider process of herpesvirus entry into host cells; insights relevant to vaccine development from *in vitro* studies of antibody-gB interactions and effects; and the results from previous *in vivo* studies using gB-based vaccines. We conclude by critically appraising the potential for gB antigens to contribute to future herpesvirus vaccines, as compared to alternative or complementary approaches.

## 1 Introduction

The human herpesviruses (HHVs) are double stranded DNA viruses (~130–250 kbp) with a common underlying architecture. The DNA genome is encapsulated within an ordered icosahedral capsid, which in turn is surrounded by a membrane-bound, protein-filled tegument (1). Envelope glycoproteins bind to surface receptors on the host cell and facilitate virion fusion (2).

To date, eight HHVs have been identified (3), all with near-obligate human tropism, while certain simian herpesviruses cause rare zoonotic infections (4). Most HHV infection is asymptomatic or causes mild symptoms, but the viruses establish life-long infection and can remain latent within infected cells for decades before reactivating. In a minority of cases, reactivation can result in significant consequences (5–8).

Despite decades of research, VZV remains the only HHV for which vaccines have been licensed. A live-attenuated vaccine against VZV is administered to infants to protect against varicella (chicken pox) and given at a higher dose to adults to protect against reactivation (herpes zoster, or shingles) (9). More recently, an adjuvanted subunit vaccine based upon VZV gE protein (VZV’s most abundant surface glycoprotein, important in immune evasion, receptor binding and cell-to-cell spread (10)) has achieved higher levels of efficacy against shingles (11), demonstrating the potential of rationally designed vaccines targeting HHV glycoproteins.

There are significant unmet needs for vaccines against other HHVs: against EBV, due to its role in the development of multiple sclerosis and cancers (12, 13); against HCMV as the leading cause of congenital disability (14); and against HSV-1 and -2 as a common cause of sexually transmitted infections (with HSV-2 acting as a co-factor in HIV acquisition) (15–17).

Most efficacious viral subunit vaccines induce antibody against virion surface proteins responsible for cell entry. For enveloped viruses, the commonest target is the viral fusion protein. Fusion-protein-based products have achieved clinical efficacy against targets including SARS-CoV-2 (18), respiratory syncytial virus (19, 20) and influenza (21, 22). In many cases, the fusion protein is also responsible for receptor binding and is highly susceptible to neutralising antibody. Some viruses, including herpesviruses, have separate receptor-binding or accessory proteins. The high efficacy of vaccination using VZV’s receptor-binding protein gE demonstrates that these too can be good vaccine targets. Herpesvirus vaccine developers might therefore be considered ‘spoilt for choice’ regarding possible targets. Unlike simpler viruses, it is not immediately obvious which essential antigen is the most promising target.

Other recent reviews have provided comprehensive overviews of developmental HHV vaccines and candidate antigens (2, 23–30). Among these, multiple vaccine candidates (particularly targeting HSV-1/2 and HCMV) have been based on gB, alone or in combination with other antigens. **Table 1** summarises selected gB-containing vaccine candidates, primarily those which have reached clinical trials with efficacy readouts. **Supplementary Table 1** provides a more comprehensive summary of gB-containing candidates which have reached any stage of clinical trial.

**Table 1:** Selected vaccine candidates against HCMV and HSV-1/2 that have progressed to clinical trials.

Virus antigen	Developer, vaccine, platform, clinical phase	Evidence for immunogenicity and/or efficacy	Ref
HSV-2 gB & gD	Chiron. Subunit with MF-59 adjuvant Phase II/III	Reactivation-prevention trial in adults with recurrent genital herpes, and two acquisition-prevention trials in healthy HSV-2 seronegative adults. No significant efficacy on primary endpoints, though some suggestions of efficacy in additional analyses (e.g. reduced severity of reactivation and reduced acquisition rate in early months after vaccination). Glycoprotein-specific and neutralising antibodies induced but did not correlate with protection.	(31, 32)
HCMV gB	Sanofi/Chiron. Subunit with MF-59 adjuvant Phase II	Efficacy against HCMV acquisition 50% (p =0.02) in recent mothers and 43% (p=0.20) in adolescent females. Efficacy correlated with antibody to cell-associated gB. In transplant recipients, neutralising antibody induced only in seropositive recipients. Duration of viraemia reduced in seronegative recipients with seropositive donors (secondary analysis, p=0.048), and correlated with anti-gB titre, but without significant neutralising antibody induction in this population.	(33–36)
HCMV gB and pp65	Vical. DNA & CRL1005 poloxamer/benzalkonium Phase III	After significant efficacy was seen on some endpoints in a Phase II trial, a Phase III trial in seropositive hematopoietic cell transplant recipients showed no significant reduction in composite endpoint (all-cause mortality and adjudicated HCMV end-organ disease, 1 year post-transplant). No detectable induction of anti-gB antibodies.	(37)
HCMV gB & pentamer	GSK. Adjuvanted subunit vaccine. Phase I/II	Immunogenicity study completed in 2025. Results expected 2026. Little information available regarding adjuvant or development plans; included here as a notable candidate given developer’s strength in the field.	(38)
HCMV gB & pentamer.	Moderna. mRNA/LNP Phase III	mRNA-1647. Induces both anti-gB and anti-pentamer antibody responses in seropositive and seronegative participants. Lower anti-gB IgG responses and lower ADCP activity compared to gB/MF59, but higher ADCC activity. Phase III study in healthy seronegative adults: reportedly 6 to 23% efficacy against primary infection (non-vaccine HCMV antigen seroconversion), leading to programme termination. Separate Phase II study with efficacy endpoints in hematopoietic stem cell transplant patients expected to read out in 2026.	

This table focuses primarily on candidates for which efficacy data is available. **Supplementary Table 1** provides a more comprehensive listing of clinical trials of gB-containing vaccines, including additional candidates from Alphavax, Hookipa, VBI and GSK. To our knowledge no gB-based vaccines against EBV or VZV have entered clinical.

Here we review gB’s structure and function, the nature and protective effect of immune responses to the protein, and the development of gB-containing HHV vaccine candidates. We conclude by reviewing key uncertainties facing developers of gB-based vaccines, regarding the antigen’s attractiveness relative to other potential targets, the importance of its conformation, and the immune effector mechanisms which developers should be seeking to induce.

## 2 gB structure and function

### 2.1 Structural overview

The structural fold of gB is conserved across the herpesviruses and has similarities with other class III viral fusion proteins (e.g. those of rhabdoviruses and baculoviruses) (39). Like Class I proteins (such as HIV env or flu haemagglutinin), they form homotrimers in both their pre- and post-fusion states, with a central bundle of α-helices (39). Their β-sheet-rich fusion domains, however, more closely resemble those of Class II proteins (such as dengue virus E) (40).

**Figure 1:**
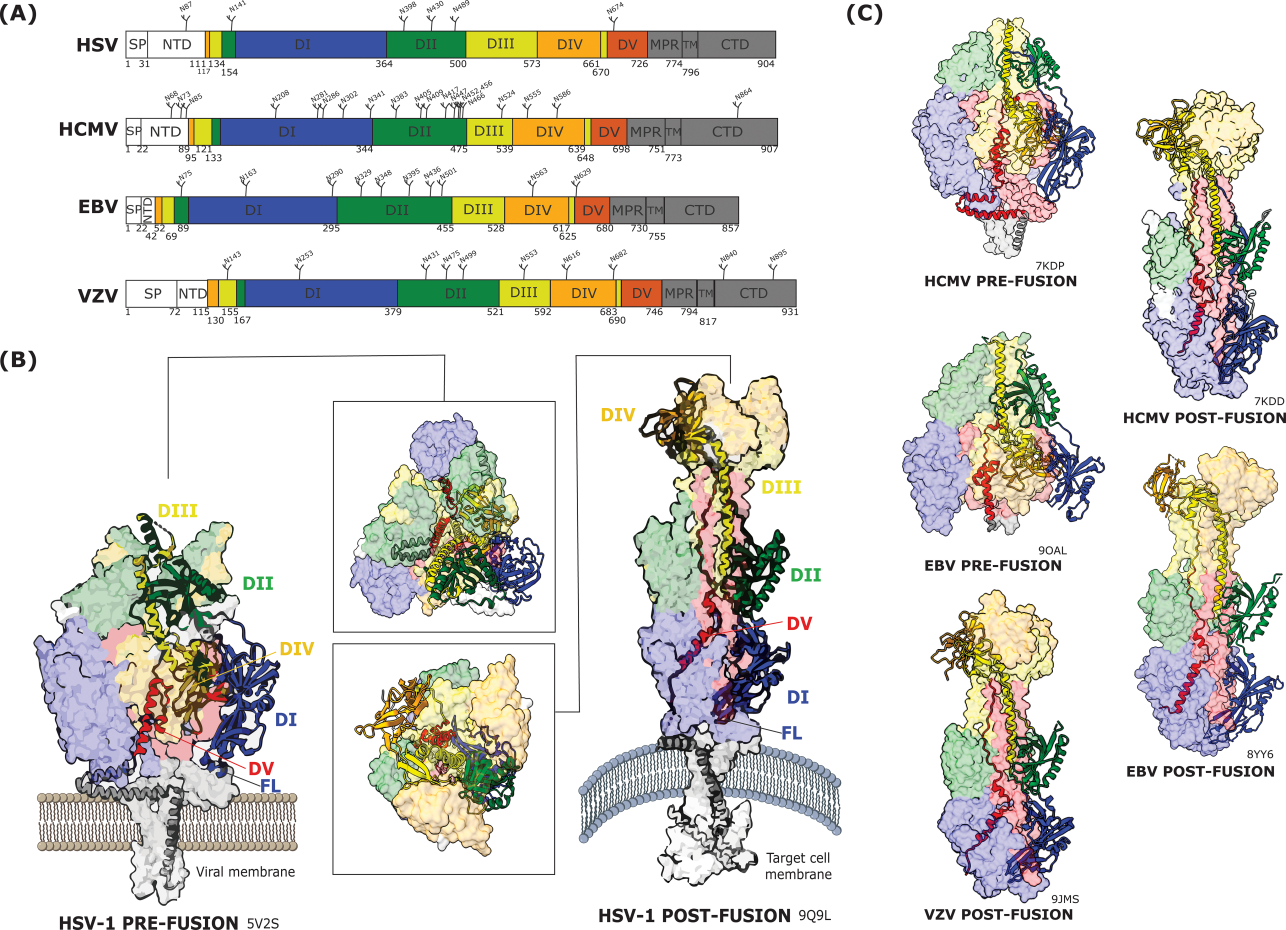
Overview of herpesvirus gB architecture. **(A)** Schematic of domain layout within primary sequence of HHV gB. Sequences are shown for HSV-1, HCMV, EBV and VZV. SP = signal peptide; NTD, N-terminal domain (white); DI, fusion domain (blue), DII, pleckstrin homology domain (green); DIII, central helix (yellow); DIV, post-fusion crown (orange); DV, linker (red); MPR, membrane proximal region (grey); TM, transmembrane domain (grey); CTD, C-terminal domain (grey). Trees represent position of predicted N-glycans. The numbering corresponds to the starting position of each domain block, per literature consensus. **(B)** Structure of trimeric HSV-1 pre-fusion ectodomain and CTD (left, PDB 5V2S (41)) and full-length post-fusion gB (right, PDB 9Q9L (42). Domains are coloured as per **(A)** and shown in relation to manually added membrane to assist visualisation, with cartoon representation of one protomer and surface presentation of the others. Fusion loops, on the tip of DI, are labelled FL. Insets show top views of the respective conformations. **(C)** Published structures of gB for selected other HHVs, with depiction and domain colouring as for **(B)**. Pre-fusion structures are shown for HCMV and EBV (PDB 7KDP (43) and 9OAL (44) respectively). Post-fusion structures are shown for HCMV, VZV and EBV (PDB 7KDD (43), 9JMS (45) and 8YY6 (46) respectively). To our knowledge no high-resolution structure of pre-fusion VZV gB has yet been reported.

Herpesvirus gB proteins share five extracellular domains, DI to DV (**Figure 1**). In the primary sequence, DI, the fusion domain, is central. DII-IV are arranged ‘palindromically’ around DI. DII contains a pleckstrin homology domain. DIII comprises the central helix involved in trimerisation. DIV lies at the base of the post-fusion conformation but forms the membrane-distal ‘crown’ of the protein in post-fusion conformation. DV forms a long ‘linker’ from DIV to a membrane-proximal region (MPR) which stabilises the fusion loops by protecting them from the aqueous environment (41, 42). The protein is anchored in the envelope by a single-pass transmembrane domain (TMD), which extends into an intravirion/cytoplasmic C-terminal domain (CTD) (41, 47).

### 2.2 Triggered transition from pre- to post-fusion conformation

**Figure 2:**
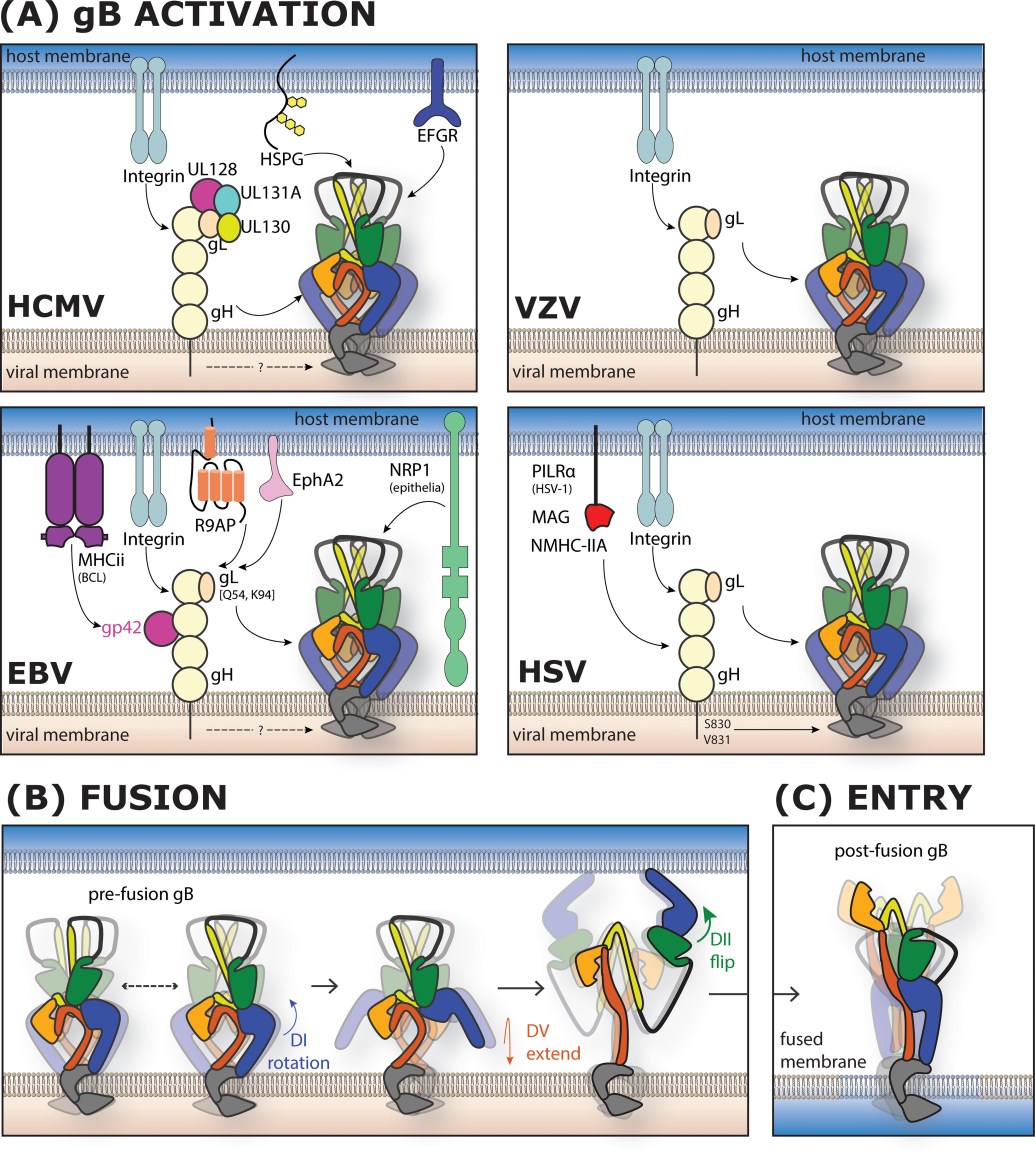
Mechanisms of herpesvirus gB-mediated membrane fusion. **(A)** gB serves as downstream effector of receptor-binding and regulatory proteins, as shown here for VZV, EBV, HSV and HCMV. The heterodimer gHgL serves as a conserved activator of gB. **(B, C)** The pre-fusion state shifts between an open and closed conformation, during which the DI (with fusion loops) swings outwards. Subsequently DV can ‘extend’, allowing the fusion loops to embed in the target cell membrane. To draw the host-membrane and virion membrane together (fusion, and hence entry), DII swings outwards, driving in membrane fusion and resulting in the stable post-fusion structure with DI once again close to DV, the MPR and TM domain. Colouring as for **Figure 1**, i.e. DI blue, DII green, DIII yellow, DIV orange, DV red, MPR & TM grey.

Across the herpesvirus family, core elements of the cell entry apparatus are conserved. Although there is evidence for some gB proteins interacting directly with host receptors (48–51), gB is not itself regarded as the key receptor-binding protein. Instead, gB drives membrane fusion as the downstream ‘effector’ of other proteins which mediate receptor binding, control and triggering of gB (**Figure 2**).

The gH/gL heterodimer is the other conserved core element of this apparatus, acting as the master regulator of gB (52). In some instances, gH/gL itself acts as a receptor-binding protein, but each HHV also has one or more additional species-specific receptor-binding glycoproteins (**Figure 2A**). Of these, HSV gD and its EBV analogue gp42 are essential, as are gO, UL128, UL130A and UL131 in HCMV (39, 52). The interaction of these receptor-binding proteins with host receptors determines viral tropism (53). For some HHVs entering some cell types, endosomal acidification is required – in addition to receptor binding – to trigger entry (54).

The mechanism by which the receptor binding signal is transduced by gH/gL to activate gB has recently been reviewed but is not fully understood (55–58). In HCMV and HSV-1, in the absence of receptor binding, gB and gH/gL appear to exist as a constitutive complex (59–61). It has been suggested that a conserved glycan-free face on gH may interact with gB’s ectodomain, and that inhibition of this interaction by anti-gH mAbs binding this face may prevent normal gB function (58). In contrast, it is proposed that, after receptor binding, the C-terminal domain of gH may activate gB, triggering fusion by acting as a ‘wedge’ disrupting an inhibitory ‘clamp’ formed by gB’s intravirion C-terminal domain (62, 63).

Understanding of gB’s conformational transition was for many years hindered by the lack of high-resolution description of the structure of pre-fusion gB proteins. Solving these structures proved challenging due to difficulty in preparation of stable pre-fusion gB, partly because of the importance of the MPR, TMD and CTD in stabilising this state. Pre-fusion structures of multiple HHV gB proteins have recently been elucidated, complementing previously described post-fusion structures (**Figures 1B, C**) (41, 64–66). HCMV gB was the first family member for which paired high-resolution pre- and post-fusion ectodomain structures were available, with elucidation of the former requiring chemical stabilisation (a combination of a fusion-inhibiting small molecule binder and chemical crosslinking) (43). More recent reports have described mutational stabilisation of multiple gB proteins, involving a combination of stapling domains, introduction of prolines and cavity filling, including those of HCMV, EBV, HSV-1 and VZV (42, 44, 67, 68).

A sequential process of architectural rearrangement of gB is then hypothesised to result in pre- to post-fusion conformational change, driving membrane fusion: **Figure 2C** shows a recent model for this (67). Notably, and in contrast to the pre- to post-fusion rearrangement of Class I fusion proteins, the central helix of gB remains relatively unchanged during this conformational transition. Nor does the transition involve major alteration to secondary structure elsewhere in gB. Instead, folded domains pivot around each other en bloc, on flexible linkers. The resulting post-fusion conformation is highly stable and the favourable thermodynamics of this series of structural changes provides the necessary energy to overcome the kinetic barrier to membrane fusion (69).

## 3 Immunology of gB

### 3.1 Antigenic landscape and neutralising antibodies

Across herpesviruses, gB is immunogenic in the context of natural infection. The relative abundance of gB protein in virions and the presence of anti-gB antibodies in serum from infected people contributed to gB being among the earliest viral proteins to be identified for each species (although not initially named as such). It has not been proven whether this naturally acquired anti-gB response has a meaningful protective effect. Most studies have found that anti-gB antibodies contribute a minority of total naturally acquired neutralising antibody. Instead, such antibody is predominantly directed to receptor-binding proteins including gH/gL-based complexes such as HCMV pentamer (65, 70, 71), EBV gp350 (72), HSV gD (73) and VZV gE (74).

The general pattern of limited neutralisation by naturally induced antibody to gB (as compared to that against receptor binding proteins) is mirrored in studies of vaccine-induced antibody. Although anti-gB typically has broader effects upon entry to a wider range of cell types, receptor-binding protein antibodies induce very high neutralisation titres when assayed on favourable cell types, exceeding those typically induced by gB immunogens (71, 75–77). Some studies are exceptions to this pattern, reporting good neutralising potency of anti-gB antibody after vaccination (78, 79) and in infected individuals (80). Interestingly, it has been reported that mixing vaccine-induced anti-gB and anti-gH/gL-complex antibodies can achieve what appear to be truly synergistic neutralising effects (i.e. supra-additive) (79).

Numerous anti-gB monoclonal antibodies (mAbs), both neutralising and non-neutralising, have been isolated and characterised. In keeping with gB’s essential role as a fusion protein, neutralising anti-gB antibodies (nAbs) typically block infection of all target cell types – for example B cells and epithelial cells for EBV (81), or epithelial cells and fibroblasts for HCMV (82, 83). However, generally potency of anti-gB nAbs tends to be weaker than that of nAbs to receptor-binding proteins. Anti-gB IC50 values are rarely <10 ng/mL and often >1 µg/mL; some anti-receptor-binding-protein mAbs have IC50 in the low ng/mL range – on certain cell types (74, 84, 85). It is unknown whether this may reflect a requirement for higher occupancy of epitopes to achieve neutralisation, or temporally or spatially limited accessibility of the vulnerable epitopes on gB.

**Figure 3:**
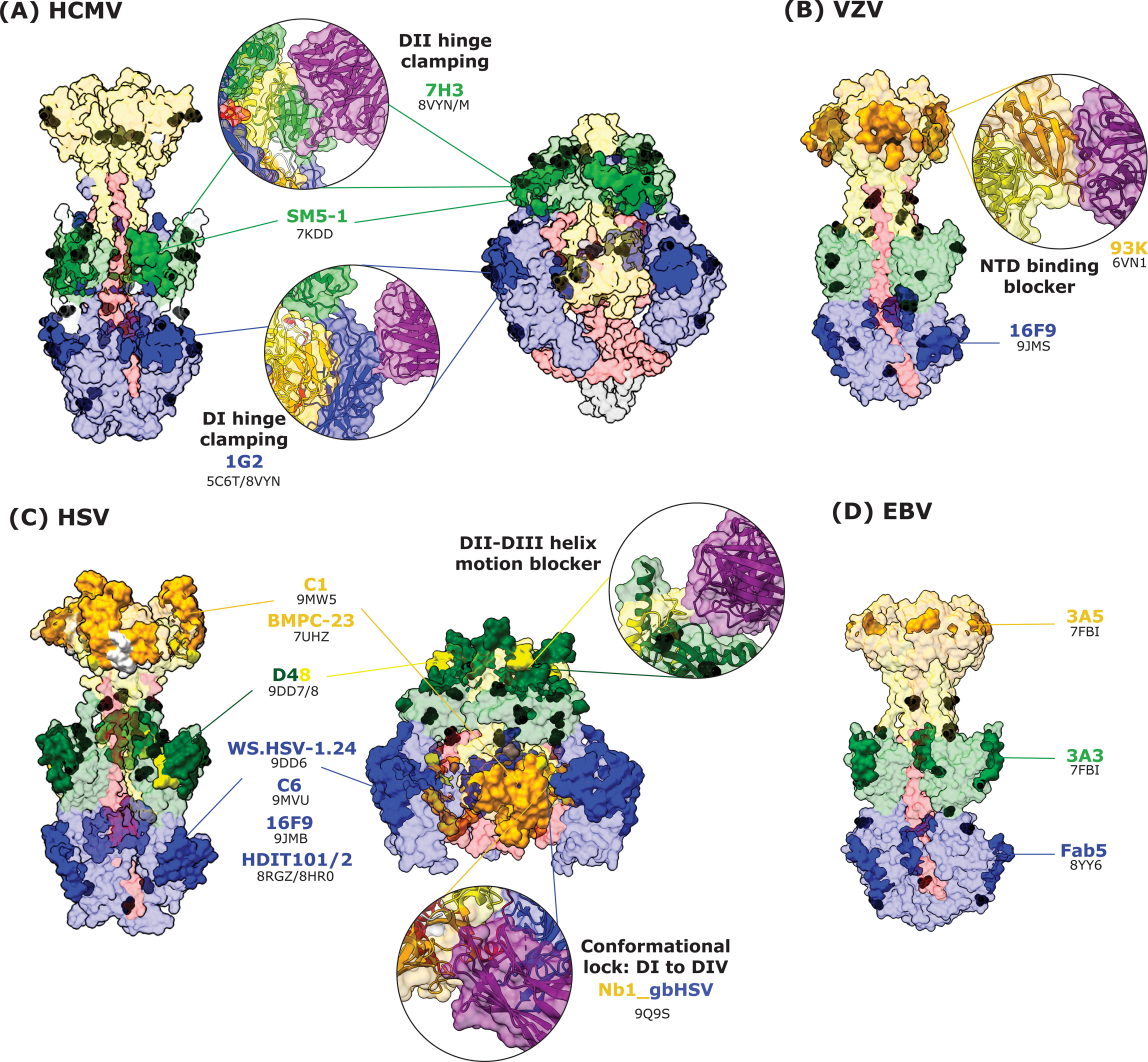
Neutralising antibody epitopes. Surfaces of gB proteins are shown, derived from the same PDB entries and coloured as in **Figure 1** (i.e. DI blue, DII green, DIII yellow, DIV orange, DV red, MPR & TM grey). nAb contact residues were defined those < 4Å from nAb residues in nAb:gB complex PDB entries. Contact residues are highlighted in darker shade of colour and labelled with the name of the corresponding nAb (coloured according to the main domain bound, or in some instances in two colours where two domains are bound) and PDB entry. Black balls indicate predicted N-glycosylation sites. Black text under nAb names indicates PDB entries for corresponding complexes. Insets provide closeup view of nAb: gB contacts, with nAbs shown in purple and the postulated mechanism of neutralisation stated in black text. **(A)** HCMV gB post- and pre-fusion, with footprints of nAbs 1G2, 7H3 and SM5-1. **(B)** VZV gB post-fusion, with footprints of nAbs 16F9 and 93K. **(C)** HSV-1 gB post- and pre-fusion, with footprints of nAbs C1, BMPC-23, WS.HSV-1.24, C6, 16F9 and HDIT101/2. Some of these nAbs target HSV-2 but are shown on the HSV-1 structure for conciseness. **(D)** EBV gB post-fusion, with footprints of nAbs 3A5, 3A3 and Fab5.

Distinct antigenic sites on gB proteins have been described for around 30 years (86). There is now good understanding, including from structural studies, of the binding footprints of multiple antibodies on the surface of pre-fusion and post-fusion conformations of both HCMV and HSV1/2 gB. More limited information is available regarding the antigenic landscape of EBV and VZV gB (**Table 2**; **Figure 3**). Species-specific nomenclature for sites on HCMV gB and HSV1/2 gB is sometimes used: in the case of HCMV, ‘Antigenic Domains’ (AD (86)), in the case of HSV1/2 gB, ‘Functional Regions’ [FR (87)]. **Table 2** shows the relationship between this species-specific epitope nomenclature and structural domains (DI – DV, as shown in **Figure 1**).

**Table 2:** Antibody epitopes.

Structural domain	Comments	Neutralising and non-neutralising mAbs known to bind each domain (with species-specific antigenic locus nomenclature for HCMV and HSV)			
		EBV	HCMV	HSV-1/2	VZV
N-terminal domain (NTD)	Mostly unresolved in published structures. Subdivided into two sites in both HCMV and HSV. Helical portion (HCMV residues 67-86, HSV residues 86-110, and VZV residues 100-116) resolved by McCallum & Veesler, contacting DI hinge in pre-fusion and DIV in post-fusion (67). In HCMV, an antigenically conserved site (AD2 site 1, residues 68-77) is recognised by neutralising antibody. An antigenically variable site (AD2 site 2, residues 50-54) within a wider region of sequence heterogeneity across the five main gB genotypes (residues 26-70) is recognised by non-neutralising antibody (88, 89). NTD-binding neutralising antibodies e.g. HCMV 3–25 may inhibit the interaction of DIV with the N terminus (67)		(AD2) Site 1: ITC88 (90), 3-25, TCN202 and TRL345 (82, 91), C23 (88).	(FR4) H1817 (aa31-43) (87)	
DI (with fusion loops) +DV	Heavily glycosylated in HCMV (four N-linked glycans). Immunisation with HCMV DI (AD5) elicits nAb in mice (92). Some neutralising antibodies (e.g. HCMV AD5 nAb 1G2 and HSV1/2 WS.HSV-1.24) bind near DI-DII hinge (obstructing which may inhibit transition to post-fusion), distant from fusion loops. A cluster of human mAbs isolated against HCMV AD5 (termed ‘1B03-like’) have also been shown to reduce cell-to-cell spread *in vitro* along with ADCC, ADCP and complement-dependent cytotoxicity (93). HSV1/2-neutralising nanobody Nb1_gbHSV binds across DI & DIV of adjacent protomers and is pre-fusion specific (42). The fusion loop-bearing tip of DI abuts DV and the membrane proximal region, which contribute to some nAb epitopes (as is known to be the case for some HSV FR1 mAbs); such antibodies may act by inhibition of fusion loop insertion into host membrane or by bridging domains. Many non-neutralising mAbs also bind in this area. In HCMV, a DV-derived epitope has recently been termed AD6 & vaccine-induced antibodies against this have been shown to affect cell-cell spread (94).	Fab5 (46)	(AD5) 1G2 (near hinge) (65). Also SM10 and 2C2 (95) and a cluster of human mAbs (inc. 1B03) identified as binding a separate region of AD5 to 1G2 (93) (AD6)	(FR1) H126, H233, H1375, B4 and H1435 (96) HDIT101, HDIT102, 2c (97) SS55, SS56, SS118 (87) Nb1_gbHSV (42) DV binders: DL16 (98); SS83, SS84, SS99, SS121, SS144, SS64, SS63 SS89, SS120, SS106, SS11, SS63, RC70) (87)	
DII (pleckstrin homology; pre-fusion crown/post-fusion ‘waist’)	Heavily glycosylated e.g. three N-linked glycans in HCMV. Target of neutralising antibody, which may act by multiple mechanisms: Blocking of gB interaction with gHgL (shown for HSV1/C226) (99). Hindrance of the DII-III helix transition to post-fusion conformation, ‘impeding DII flipping’ (suggested by model of HSV1/D48 binding) (67) Blockade of trimer transition between open and closed conformations (suggested by model of EBV/3A3 binding) (67) HSV1 pre-fusion-specific nanobodies Nb2-4_gBHSV bind DII helix αX and are pre-fusion specific but non-neutralising (42).	3A3 (81) AMMO5 (100) 8A9, 8C12 (101)	(AD4) 7H3 (AKA LJP538), SM5–1 family (near DI hinge, includes SM1–6, SM3-1, SM4–5, SM6–5 and SM11–17) (65, 68, 102, 103)	(FR2) D48 (46, 67) C226, H1781, H1838 (the latter two only partially neutralising) (87, 99) Nb2-4_gBHSV (42)	
DIV (pre-fusion ‘base’, post-fusion crown) +/- DIII (central helix)	Previously reported as an immunodominant site on basis of frequency of recognition by naturally HCMV infected individuals (102, 104) and in HCMV gB/MF59 vaccinees (though these studies did not assess AD3 (105). Contains some neutralising epitopes but many antibodies to this site are poorly neutralising (e.g. in one HCMV panel, only 2% of AD1 binders found to be neutralising (102, 106). HSV-2 non-neutralising antibody BMPC-23 induces ADCC and protects mice against challenge, while HSV010–13 does neither (107).	3A5 (81)	(AD1) EV2038 (108). Large non-neutralising panels (102, 106).	(FR3) ITC48, ITC52, ITC63B, ITC63C, 7-17, B2, B5 (96) SS10, SS67, SS68, SS69 (87) BMPC-23, HSV010-13 (107).	93k SG2 (109)
Intravirion Domain	Not recognised by any neutralising antibodies. Suggested to be (unhelpfully) immunodominant in gB/MF59 vaccinees and in rabbits immunised with a full-length gB-encoding mRNA vaccine when screening against linear epitopes only (25, 40).		(AD3)		

Relationship between structural domains for HSV and HCMV gB with their functional regions (FR) or antigenic domains (AD), respectively (86, 87) and summary of selected known mAbs against five HHV gB proteins.

As shown in **Table 2**, there are numerous examples of neutralising antibodies binding to the flexible N-terminal domain, DI (fusion domain), DII (pleckstrin homology domain) and DIV (the pre-fusion base & post-fusion ‘crown’). For HCMV and HSV1/2, there are examples of nAbs binding all four of these major regions. Epitopes of nAbs against EBV gB have been mapped in DI, DII and DIV while for VZV, to our knowledge, only one anti-gB nAb has been structurally characterised, binding DIV (**Table 2**). Recent reports of multiple pre-fusion gB:mAb complex structures have added considerably to understanding of mechanisms of neutralisation. Postulated mechanisms are summarised in **Table 2** and illustrated in **Figure 3**.

Antigenic variability is an important consideration for the design of fusion-protein-targeting vaccines and antibodies. Neutralising-antibody-sensitive Class I and Class II fusion proteins of RNA viruses and retroviruses (for example those of HIV-1, influenza, SARS-CoV-2 and dengue viruses) provide classic examples where high levels of within-species variability pose major challenges. By comparison to these examples, HHV gB proteins are generally highly conserved within species. Notably, 99% of amino acid residues in EBV gB and 92% of residues in HCMV gB are essentially invariant (by-residue Shannon’s entropy <0.1 across >200 clinical isolate sequences)^1^ (110–112). This is despite HCMV gB being considered hypervariable by comparison to other HCMV genes (111), with combinations of three multi-allelic regions resulting in twelve identified haplotypes. Studies of sera from gB/MF59 clinical trial participants have found suggestions of a functional effect of this variation only in a limited number of assays/contexts (e.g. heterologous strain neutralisation was seen in the healthy young adult Phase I population, but not in post-partum Phase II participants) (113). There is, to our knowledge, no evidence that the limited variability in EBV gB is of immunological importance.

Considering variability between species, there is (as would be expected) progressively greater sequence and antigenic divergence between-species but within-genus, between-genus but within-subfamily, and between subfamilies. Between certain within-genus pairs (e.g. the simplexviruses HSV-1 and HSV-2 (114, 115), the human lymphocryptovirus EBV and non-human relatives such as rhesus lymphocryptovirus (101, 116)), high levels of sequence conservation (>80% pairwise amino acid identity) result in considerable antigenic conservation, such that cross-neutralisation by polyclonal serum is common and many monoclonal antibodies are cross-reactive. Between genera within the same subfamily, there are limited reports of cross-reactive antibodies; such as between gammaherpesviruses EBV and KSHV (117, 118) and alphaherpesviruses HSV-1/2 and VZV, albeit with measurable differences in affinity (45). Between subfamilies, sequence divergence is greater and there is very little data suggesting cross-reactive gB-directed antibodies could have any biologically meaningful effect (119).

Structural studies have helped to reconcile the contrast between a high level of conservation of the gB structural fold and the relative rarity of broadly reactive neutralising antibodies.Functionally, conserved residues are predominantly located within the internally shielded parts of the trimer, reducing the accessibility for antibody binding (120). Whilst nAb footprint mapping identifies structurally analogous sites of vulnerability, these surface-exposed antibody-contact residues within these sites are often more variable and subject to differential glycan shielding (121). For example, a pre-fusion-specific human HSV gB neutralising-mAb targets the DI/II hinge region, which is otherwise occluded by glycans in HCMV or EBV, limiting cross-reactive antibody recognition (114). Together, these findings suggest that, whilst structural conservation of gB is extensive, antigenic accessibility is shaped by differences in epitope exposure and glycan shielding between HHVs, presenting a major challenge for the elicitation of broadly neutralising or pan-HHV antibody responses through vaccination.

Experiments using mAbs in passive transfer – infectious challenge experiments have demonstrated repeatedly that antibodies capable of *in vitro* neutralisation of a given virus can confer protection against that virus, with examples spanning all subfamilies (alpha, beta, gamma) (115, 118, 122). Frequently, however, such experiments have used Fc-receptor-engaging IgG subclasses and hence do not show conclusively that Fc-receptor-independent neutralisation is necessary and sufficient for protection. The following section describes the (sometimes surprising) results of experiments which have attempted to dissect the contributions to protection of neutralisation and Fc-dependent effector functions.

### 3.2 Fc-dependent & other non-neutralising functions of anti-gB antibodies

There are multiple strands of evidence suggesting that Fc-dependent and other non-neutralising anti-gB antibody effector functions may contribute to protection. Such mechanisms can be broadly divided into complement-mediated mechanisms, Fc-receptor-mediated mechanisms, and effects upon cell-to-cell spread.

#### 3.2.1 Complement

The multiple mechanisms of complement action against viruses, and viral immune evasion of them, have been reviewed elsewhere – both for viruses in general (123–125) and in the specific case of HCMV (126). Complement-dependent (or complement-enhanced) neutralisation is well-described for antibodies to HCMV and HSV-1 gB, including monoclonal antibodies, polyclonal antibody induced in animals by pre-clinical vaccine candidates, and serum from HCMV gB/MF59 clinical trial participants (127–130). In some cases, notably gB/MF59 clinical trial sera and sera from rabbits vaccinated with a similar commercially-available antigen, this effect can be substantial (e.g. c. 10-fold increase in titre (127, 128)). This is likely to involve deposition of C1q on the Fc of virus-bound antibodies (the first step in the classical complement pathway). Whether this acts through enhanced steric hindrance of gB function, viral aggregation, or through membrane-attack-complex (MAC)-mediated virolysis has not, to our knowledge, been defined (124). In contrast, HCMV pentamer-specific antibodies are commonly potently neutralising in the absence of complement (84, 131).

Antibody-dependent complement-dependent cytolysis (AD-CDC - i.e. lysis by the MAC as an effector of classical pathway activation by C1q binding to antibody) has long been recognised as a mechanism of action of anti-herpesvirus antibodies, as have viral mechanisms to subvert it (132, 133). AD-CDC specifically by antibodies to gB has been reported for HSV-1 gB (134). There is less knowledge regarding this mechanism for other herpesviruses. This may simply reflect the lack of specific investigation (given the relatively complex assays involved), or particular importance of AD-CDC for HSV-1/2 (which, notably, have evolved means of subverting downstream complement components via the gC-1 C3-binding protein (134)). Conversely, it may reflect a true lack of activity due to *more* effective MAC evasion by other herpesviruses, for example because of the upregulation by EBV and HCMV of host complement regulatory factors such as CD46 and CD55 (125, 135).

#### 3.2.2 Fc-receptor-dependent cell-mediated mechanisms

The principal Fc-receptor-dependent (and hence effector cell-mediated) mechanisms of antiviral antibody action are antibody-dependent cellular phagocytosis (ADCP) and antibody-dependent cellular cytotoxicity (ADCC). Both the importance of these mechanisms and their subversion by viral Fc-receptor evasins is well established for HCMV and HSV-1/2, and have recently been reviewed in detail (136). We will concentrate here on evidence specific to anti-gB antibody.

Protection of mice against HSV-2 by a non-neutralising anti-gB mAb (non-nAb) was demonstrated over 40 years ago (137): this mAb induced antibody-dependent cell-mediated cytotoxicity (ADCC) *in vitro* and retained its protective capability in mice deficient in C5 (and hence lacking complement-dependent virolysis and cytolysis capability). Similar findings have been recapitulated and extended upon in at least two further studies (107, 138). Another recent report has shown that protection by anti-gB mAbs in a murine HSV-1 challenge model is markedly reduced by ‘Fc-silencing’ mutations and (as previously) poorly predicted by neutralising potency (139). Interestingly it has been suggested that the epitope of the HSV-protective anti-gB non-nAb BMPC-23, which activates human FcγIIIa and ADCC, may be accessible only on *post*-fusion gB (107). Synergistic *in vivo* protection against HSV-2, again incompletely predicted by *in vitro* neutralisation, has also been reported for a pair of anti-gB mAbs capable of inducing antibody-dependent cellular phagocytosis (ADCP) but not ADCC or CDC (97). Similarly, both nAb and non-nAb against murine CMV gB were able to protect against lethal challenge *in vivo*, although their effector mechanism was not determined (122).

Correlations between non-neutralising anti-gB antibody and protection against HCMV and HSV have also been reported in both observational and vaccinated clinical cohorts (136).

A study of HCMV^+^-donor, HCMV^--^recipient lung transplant patients has shown that strong ADCP responses are induced post-transplant, with control of infection correlating with anti-gB IgG3 titres (140). ADCP, ADCC and anti-gB responses to maternal infection have each been shown to correlate with protection against congenital HCMV (141, 142). In this context, it appears ADCC may be the best single predictor of protection identified to date. Importantly, however, recent evidence implicates non-structural proteins (notably the NK-cell evasin UL16) as the major target of naturally acquired anti-HCMV ADCC activity, rather than gB (142, 143).

The gB/MF59 HCMV vaccine achieved c. 50% efficacy across trials (summarised in **Table 1**), despite failing to induce neutralising antibody in most assays (though see above regarding complement enhancement) (113). In adolescents and post-partum women, this protection correlated with antibody binding to cell-associated gB (and the gB immunogen), rather than any measurable effector function (144). Similarly, in gB/MF59-vaccinated solid-organ transplant recipients, outcome was associated with binding antibody titres to gB and AD2 (the NTD), while ADCC could not be detected (33, 105).

There has been considerably less study of Fc-receptor-dependent mechanisms of antibody action against VZV or EBV. This is of particular importance in the case of EBV, due to the lack of licenced vaccines, but understanding is hindered by the limitations of available animal models and the fact that clinical trials of EBV vaccines have, to date, focused upon gp350 and gH/gL/gp42 (selected for neutralising antibody induction). As discussed with respect to AD-CDC, lack of knowledge may partly reflect the effectiveness of viral subversion mechanisms. For example, it has been shown that CD55 overexpression induced by EBV latency membrane protein 1 (LMP-1) effectively inhibits ADCC by the anti-self mAb cetuximab – it seems likely it would have a similar effect upon ADCC induced by antibody to a viral protein (135).

AD-CDC and ADCC (but not ADCP) require binding of antibody to cell-surface viral antigen. Given this, it is worth noting that HSV, HCMV and EBV all undergo envelopment and cellular egress preferentially into intracellular organelles, such as the trans-Golgi network, rather than at the cell surface (145). This is accompanied by preferential retention within intracellular membranes of envelope glycoproteins, including gB, by ER retention signals and the action of evasins such as HCMV UL141 (146). This is, however, relative rather than absolute retention – gB remains present in the plasma membranes of lytically-infected cells (147) – evasion of effects of anti-gB against infected cells may similarly be relative rather than absolute.

To summarise, there is reasonable evidence (in the case of HCMV & to a lesser extent HSV), for a contribution to protection of anti-gB-induced ADCP of virions. While there is increasing evidence of the importance of ADCC in immunity to herpesviruses (particularly HCMV), it is less clear that this results specifically from antibody to gB.

#### 3.2.3 Antibody-mediated inhibition of cell-to-cell spread

A further mechanism of antibody-mediated protection – distinct from both neutralisation and FcR-mediated functions – may be the inhibition of direct cell-to-cell spread. Again, this has been most extensively studied in HCMV and HSV. Cell-to-cell spread requires membrane fusion by a process likely similar, but not identical, to primary cell entry (148). For HCMV, this appears to be the dominant mode of dissemination when subculturing clinical virus isolates (149–151).

Whilst this mode of viral transfer is reliant on gB (152–154), subcellular localisation of the antigen may limit access by antibody (as compared to the accessibility of gB on the virion) (155). Indeed, there is evidence for HSV-1, HSV-2 and HCMV showing that neutralising anti-gB antibodies cannot necessarily inhibit cell-to-cell spread if administered after primary infection (148, 149, 156, 157). There are, however, examples of anti-gB nAbs that can inhibit direct cell-to-cell spreading of HSV-1/2 (158) and HCMV (91, 108, 159) and one example of a *non*-neutralising monoclonal antibody against HCMV gB, which suggest that such spread-inhibiting anti-gB antibodies may be distinct in specificity from anti-gB nAbs, and rare in natural infection but common in gB/MF59 vaccinees (94).

EBV can propagate in B cells without cell-to-cell spread (by driving proliferation), but cell-to-cell spread may nonetheless be of importance in EBV infection of epithelial cells (160–162). As far as we are aware there is little understanding of how this may be affected by vaccine-induced antibody.

### 3.3 T-cell responses to gB

The central importance of cell-mediated immunity in control of herpesvirus infection is well known, and clearly illustrated by the vulnerability to herpesvirus of individuals with deficiencies in T cell function (163).

gB-specific CD8^+^ and/or CD4^+^ T-cell responses are commonly detectable in people infected with human herpesviruses. In some contexts, associations between gB-specific T-cell responses and clinical outcomes have been observed. For example, among HSV-1 carriers, certain subsets and epitope specificities of anti-gB CD4^+^ and CD8^+^ T-cells have been shown to correlate with asymptomatic carriage (164, 165).

Much as is the case for naturally acquired anti-gB antibody responses, however, a causal relationship between gB-specific T-cell responses and clinical protection is harder to establish.

In the CD8+ T-cell response to HSV-1 infection in C57/BL6 mice the immunodominant epitope is the gB-derived peptide SSIEFARL, recognised by up to 50% of HSV-specific T-cells (166). Passive transfer of transgenic CD8+ T-cells specific for this epitope is protective against HSV-1 challenge of immunodeficient (RAG-/-) mice (167). This is perhaps the clearest example of causation of protection by gB-specific T-cells – but experience from other pathogens suggests considerable caution in extrapolating to humans a finding of protection by T-cells against an immunodominant epitope in mice (168).

Taken together, it seems reasonable to think that gB-specific T-cells may contribute – alongside antibody, and T-cell responses to the multiple other viral proteins known to elicit strong T-cell responses – to control of HHV infection, either after natural infection or vaccination. It is debatable whether evidence of control by this mechanism is sufficiently strong to motivate a vaccination strategy prioritising T-cell responses to gB over other possible approaches (including T-cell targeting vaccines against intravirion and intracellular antigens). As is the case for humoral responses, the many unanswered questions regarding the causation of protection by anti-gB T cell responses highlight the need for efficacy data from clinical trials of diverse vaccine candidates.

## 4 Clinical experience with gB-targeting vaccines and mAbs

There is a history of more than 35 years of vaccine development targeting HHV gB proteins. Candidates which have reached clinical trial are described in **Supplementary Table 1**. **Table 1** in the main text shows abbreviated information, primarily for candidates for which efficacy data is available.

gB-based vaccine candidates reaching clinical trial have, to our knowledge, exclusively targeted HCMV and HSV-1 and -2. These have achieved, at best, partial success. Interpretation of weak efficacy results from clinical trials can be complex, but the repetition of partial efficacy signals across multiple trials appears somewhat encouraging.

The most notable success has been achieved by the HCMV gB recombinant protein/MF59 adjuvant vaccine developed by Chiron and latterly Sanofi. As summarised in **Table 1**, this achieved efficacy signals in Phase II trials in three different populations. Associations of this efficacy with non-neutralising antibody induction have been described above (Section 3.2). These encouraging results were obtained using a protein antigen designed without the benefit of the structural information now available, and believed to have presented primarily a post-fusion monomer (127).

Humoral responses have also been characterised in detail in subsequent clinical trials of two other gB-containing vaccines: Moderna’s mRNA-1647 (which also includes mRNA encoding the receptor-binding pentamer complex), and VBI1501A (an alum-adjuvanted enveloped virus-like particle vaccine). Efficacy estimates of 6-23% for mRNA-1647 have been seen in some quarters as disappointing (169), not least by comparison to the most similar of the gB/MF59 trials, which found 50% efficacy against a similar endpoint in a similar population (seroconversion to non-vaccine antigens in seronegative women of child-bearing age) (34). Efficacy of VBI1501A has not been assessed, but the available data regarding its immunogenicity illustrates the potential to induce qualitatively different immune responses with different presentations of gB. Valuable studies by Permar and colleagues have used samples from HCMV gB/MF59 vaccinees and naturally infected seropositive individuals as benchmarks against which to compare the immunogenicity of these later candidates (38, 170).

The pattern of these responses is complex and challenging to relate to the nature of the immunogens; f or full description, see **Supplementary Table 2**. In brief:

- Although it induced little neutralising antibody, the soluble post-fusion gB/MF59 excelled in induction of antibody binding the post-fusion protein, exceeding seropositives or subsequent products.
- mRNA-1647 presents membrane-associated full length gB in a Th1-skewing formulation, with pentamer. It induces strong neutralising antibody (probably primarily directed to its pentamer components), with detectable ADCC and ADCP responses (though weaker than in seropositives). Titres of anti-gB (binding either the ectodomain or, perhaps surprisingly, cell-associated antigen) were, however, weaker than those induced by gB/MF59 and not markedly superior to those seen in seropositive individuals. The disappointing efficacy of mRNA-1647 against a stringent endpoint (seroconversion to non-vaccine antigens) may not, therefore, be particularly informative regarding the prospects of efficacy with a gB-based vaccine capable of inducing ‘supra-natural’ levels of anti-gB antibody, ADCC or ADCP.
- It has been suggested that the gB ectodomain/VSV-G fusion protein presented by VBI1501A may be in pre-fusion conformation, on the basis that cells expressing it form syncytia. Perhaps consistent with this, it induced higher responses than gB/MF59 (and perhaps, by extrapolation, higher than mRNA-1647 or seropositive responses) in assays of binding to cell-associated gB and AD4 (DII, which is membrane-distal in the pre-fusion conformation). It induced limited neutralising antibody. The Th1/Th2 skew it induced has not, to our knowledge, been reported: although alum is classically a Th2-skewing adjuvant, another product using the eVLP/alum platform has been shown to induce a more balanced IgG subclass distribution in mice (171). Unfortunately, no efficacy study has been performed, and the developer has recently entered bankruptcy/restructuring.

Taken together, these studies do not provide clear evidence favouring gB immunogens in a particular conformation, though it does seem reasonable to prioritise Th1-skewing formulations for their capacity to induce ADCC- and ADCP-competent IgG1 and IgG3. It is interesting to note that responses to the first epitopes identified in studies of naturally acquired response in HCMV seropositives (AD1, on DIV, and AD2, on the N-terminus) appear to have been particularly poorly induced by all three of these candidates.

In the HSV-1/2 fields, the only clinical efficacy results for a gB-containing vaccine have been for Chiron products tested in the 1990s, formulated with MF59 and containing other glycoproteins (31, 172). Trials of a gB/gD formulation in HSV-2 seronegatives observed a trend to reduced HSV-2 acquisition in the months soon after vaccination (at which antibody titres were presumably highest), but no effect on pre-specified acquisition or disease modification analyses over the full 18-month follow-up period (31). This lack of clear efficacy was despite the induction of neutralising antibody titres (possibly contributed to by anti-gD) exceeding those seen in naturally infected seropositives.

Clinical trials of mAbs against HCMV and HSV-1/2 gB (given in combination with anti-pentamer mAbs in the RG7667 and CSJ148 formulations) have been summarised elsewhere (173). Rather as for vaccine studies, these present a mixed picture, with hints of efficacy in some trials on some secondary endpoints, but no unambiguous signal.

The anti-HSV-1/2 gB mAb HDIT-101 has also been evaluated in efficacy trials, again with mixed results. A trial in chronic HSV-1 participants was terminated due to futility in an interim analysis but, importantly, the trial had used only topical administration (NCT04539483). In contrast, in chronic recurrent HSV-2 participants, intravenous HDIT-101 showed possible effects of on recurrence rate in secondary analyses, while although the severity of each recurrence was similar to that observed in participants receiving valaciclovir treatment [i.e. an active comparator (174)]. These mAbs were selected primarily based on neutralisation potency (and, in the case of the anti-HCMV products, contained anti-pentamer components to further enhance epithelial and endothelial neutralisation). Notably, in these efficacy studies the doses used have been relatively high [50 mg/kg every four weeks of the anti-gB component of CSJ148 (175), three to four doses of 10 mg/kg of each component of RG7667 (176), 2 g of HDIT-101 (173)]. Consequently, the resulting serum concentrations achieved have been in the range of hundreds of µg/mL (including for the anti-pentamer component of RG7667 (177). Achieving such concentrations of antigen-specific antibody with any vaccine is likely to be challenging – let alone maintaining them for years, or decades. The failure of any of these products to achieve high level efficacy is surely sobering for the prospect of efficacy with any neutralisation-focussed vaccine strategy – whether targeting gB or receptor-binding proteins.

To our knowledge, no vaccines or mAbs targeting EBV or VZV gB have entered clinical trial. In the VZV field, this largely reflects the success of licensed live-attenuated and gE-targeting vaccines. EBV vaccinology, conversely, is a highly active field with no licensed product, and candidates targeting receptor-binding proteins now in clinical trials. The omission of gB from current clinical EBV candidates is perhaps noteworthy. Moderna’s EBV vaccine candidates omit gB, contrasting with the company’s HCMV candidate. The Moderna EBV candidate mRNA-1189 formulation targets exclusively receptor-binding proteins, with the addition of latency proteins as putative T-cell antigens in the mRNA-1195 formulation. We are not aware of a reason for the omission having been disclosed but it is tempting to speculate that this may have been based upon relative potency of neutralising antibody induction by the components.

## 5 Discussion

We feel there are three important uncertainties facing developers of future HCMV, HSV-1/2 and EBV gB-based vaccines. Firstly, should vaccine developers focus on neutralising antibody to gB, or are Fc-dependent effector functions of anti-gB antibodies likely to contribute substantially to protection? Secondly, given that it exists naturally as a conformationally-metastable membrane-associated trimer, how critical is antigen conformation and the accessibility of certain epitopes to B-cell receptors likely to be? Thirdly and finally, should gB be a priority target for HHV vaccine developers at all?

As discussed above, characterisation of anti-gB mAbs and vaccine-induced sera has made clear that neutralising activity, ADCP and ADCC activity can be independent of each other, and that inhibition of cell-to-cell spread appears particularly challenging.

A number of strands of evidence argue for a strategy which prioritises HCMV and HSV candidates inducing Fc-dependent effector functions: the partial success of the HCMV gB/MF59 vaccine (which induced non-neutralising antibody); the lack of high-level efficacy in clinical trials which achieved extremely high serum concentrations of candidate therapeutic mAbs against HCMV and HSV-1/2 gB (or indeed high concentrations of anti-HCMV-pentamer mAbs); and the clear evidence of the importance of Fc-dependent effector functions in murine CMV and HSV challenge models. There is considerably less evidence in the EBV field, where gB immunogens have yet to enter clinical trial and there is a lack of accessible animal models with intact Fc-dependent effector functions. gB-targeted antibody responses in other HHV, such as HHV6 and HHV8/KSHV, remain relatively unexplored. This likely reflects differences in clinical prioritisation and the lack of well-established models. More developed characterisation of gB in these viruses may help provide further insight into the antigenic landscape of gB across the HHV.

The relationship between gB antigen conformation, epitope accessibility and induction of functional antibody remains poorly understood. Disappointingly, the limited evidence so far available regarding the immune response to pre-fusion-stabilised gB immunogens suggests that they may confer quite limited benefit relative to vaccination with the post-fusion form. Pre-fusion-stabilised EBV gB elicited modestly higher neutralising titres than the post-fusion form in mice (c. 2-fold) (44). In the case of HCMV, a detailed comparison of the quantity, specificity and functionality of antibody induced by vaccination of mice with pre-fusion-stabilised vs post-fusion gB identified essentially no benefit of pre-fusion stabilisation (178). Interestingly, and perhaps surprisingly, there appeared to be some detrimental effects of pre-fusion stabilisation: reduced binding to cell-associated gB, AD1/domain IV and AD5/domain I, and reduced ADCP on one viral strain.

Comparison of HSV-1 gB pre- and post-fusion structures has suggested explanations for the limited immunological differences between conformations. Conformational transition results in minimal change in the regions which are exposed and accessible to antibody (termed ‘iso-surface exposure’). Conformation-specific exposed regions are limited to interfaces between domains, regions undergoing refolding tend to be buried, and the few non-buried domain-interface residues which undergo refolding are frequently shielded by glycans (179). Moreover, it was suggested that the rigid-body rearrangement involved in conformational transition may be relatively insensitive to blockade by antibody.

There is more encouraging evidence from studies investigating alternative approaches focused on epitope display or accessibility rather than pre-fusion stabilisation. Antibody induced by nanoparticles displaying post-fusion EBV gB induced markedly superior protection against EBV in humanised mice, as compared to antibody induced by soluble gB (180). The authors suggested this may have been contributed to by increased accessibility of a neutralising epitope in domain IV (equivalent to HCMV AD1, membrane-distal in the post-fusion form, against which antibody induction in some clinical trials has been poor). Nanoparticles displaying HCMV gB domain I (AD5) induced substantially higher neutralising titres in mice than soluble gB ectodomain (92). It remains to be seen whether similar strategies may be able to selectively enhance induction of Fc-dependent effector functions.

To summarise, arguments in favour of the inclusion of gB in future vaccine candidates and against it are presented in **Table 3**. gB thus poses something of a quandary for developers entering the field and picking antigens for subunit vaccines – the quandary being that two frequently followed ‘rules of thumb’ suggest divergent approaches. The first prioritises the induction of neutralising antibody, as this has historically been by far the most successful approach to viral vaccine development: developers prioritising neutralising antibody might de-prioritise gB relative to receptor binding proteins.

**Table 3:** Summary of factors influencing prioritisation of gB in future HHV vaccine candidate.

Against prioritisation of gB		Favouring prioritisation of gB
Neutralising antibody	Antibodies to receptor-binding proteins frequently achieve higher neutralising titres. Although the universal requirement for gB for entry to all cell types allows anti-gB antibody to neutralise entry to a broad range of cell type, its potency in doing so is limited and may be surpassed by antibodies targeting combinations of receptor-binding proteins. Trials achieving high serum concentrations of neutralising anti-gB *mAbs* – including, for HCMV, in combination with anti-pentamer mAb - have not achieved high-level efficacy.	Effect of anti-gB nAbs upon entry to all cell types. Evidence of synergistic neutralisation by combinations of anti-gB with anti-receptor-binding-protein antibodies.
Non-neutralising antibody induction	For HCMV, a strategy focusing on ADCC might prioritise non-structural cell surface antigens.	To our knowledge, no gB-based candidate inducing levels of ADCC or ADCP superior to those seen in seropositives has yet been clinically evaluated for efficacy.
Clinical data	Moderna’s mRNA-1647 HCMV candidate (including gB) achieved minimal efficacy against seroconversion in Phase III, despite having presented full-length cell-associated antigen (some of which is likely to have been in pre-fusion conformation) in a reasonably immunogenic formulation. Protection against uncontrolled lytic replication is clearly possible for at least some herpesviruses using non-gB antigen vaccines: VZV gE (against clinically-evident shingles); Moderna’s mRNA-1189 (partial efficacy against EBV salivary shedding); GSK’s EBV gp350/AS04 (possible efficacy against symptomatic mononucleosis).	gB immunogenicity of mRNA-1647 appears to have been poor by comparison to seropositives or gB/MF59 (**Supplementary Table 2**). Efficacy achieved by gB/MF59 probably remains the most promising result in the HCMV vaccine field to date. There are clear routes to improve upon this with more conformationally-accurate antigen (even post-fusion trimeric ectodomain) and potent adjuvant (e.g. VLP with saponin-based adjuvant. No gB-based candidate has been clinically evaluated for EBV.

See corresponding text sections for source references which (for brevity) are not repeated in the table.

A second ‘rule of thumb’ is that, in the absence of a robust immunological correlate of protection, partial efficacy in a clinical trial provides one of the strongest possible target validations for future developers. Partial efficacy can usually be improved upon by applying newer technologies to induce quantitatively or qualitatively superior responses to the same antigen. Developers guided by this second heuristic would be justified in prioritising gB as the only antigen which is both conserved across HHV species and supported by clinical efficacy validation (with HCMV gB/MF59). Our view is that careful review of the clinical performance of previously-evaluated gB-containing vaccines supports clinical development of improved candidates.

## Footnotes

1 Shannon’s entropy was calculated by analysis of previously published sequence data. EBV data was obtained from dbEBV and filtered on sequences from healthy donors or those with infectious mononucleosis (rather than EBV-driven malignancy), resulting in a set of 219 sequences. HCMV data comprised 229 sequences from clinical isolates. Both analyses excluded laboratory-adapted strains. A more limited analysis of 132 HSV-2 sequences collected from a single clinical cohort showed similar results (99% of residues with Shannon’s entropy <0.1).

## Supplementary material

Supplementary Table 1 Relating to **Table 1**, further information regarding vaccine candidates against HCMV gB and HSV-2 gB which have entered clinical trials.

Supplementary Table 2 Relating to Section 4, detailed characterisation and comparison of immune responses elicited in HCMV gB trials.
