# Supplementary material for: Progress and prospects for herpesvirus vaccination using gB antigens
Source: Front Immunol. 2026 May 29;17:1827628. doi: 10.3389/fimmu.2026.1827628 (PMC13260650; doi:10.3389/fimmu.2026.1827628)
Supplement: Supplementary Table 1 — Relating to Table 1, further information regarding vaccine candidates against HCMV gB and HSV-2 gB which have entered clinical trials. [file Table1.docx]

**Supplementary Table 1 - Vaccine candidates against HCMV and HSV-1/2 that have progressed to clinical trials, completed and ongoing.** To our knowledge no gB-based vaccines against EBV have entered clinical trials. The omission of gB from Moderna’s mRNA-1189 and mRNA-1195 candidate EBV vaccines contrasts with the inclusion of gB in the mRNA-1647 HCMV candidate.

| **Virus** | **Antigen** | **Developer & Vaccine Platform** | **Stage of Development** | **Evidence for Immunogenicity and/or Efficacy** | **Reference** |
| --- | --- | --- | --- | --- | --- |
| HSV-2 | HSV-2 gB, gD, gC/gE glycoprotein mixture | Chiron.  Alum-adsorbed glycoproteins extracted from HSV-2 infected chick embryo fibroblasts | Phase II | Induced neutralizing antibody and ELISA-detectable anti-gB antibody. No protection against genital HSV-2 infection. | (1) |
| HSV-2 | HSV-2 gB & gD | Chiron.  Subunit vaccine administered with MF-59 adjuvant | Phase II | In seropositives: Otherwise healthy adult men and women from 18-55 years with minimum 1-year HSV-2 infection and >3 genital lesion outbreaks. 10-19% decrease to genital lesion recurrence in vaccine group compared to placebo across 8-month follow period. Vaccine failed to meet primary end point efficacy cut-off. Vaccinated cohort showed significant reduction to days until disappearance of lesions as well as to number of days across which new lesions appeared in outbreaks. No significant reduction to viral shedding. Vaccine does boost anti-gD and anti-gB antibody titre and remains above pre-vaccination levels in 12-month follow-up but no significant correlation between anti-gD or anti-gB titre with improved clinical outcome.  In seronegatives: Two separate trials of HIV-, HSV-2 doubly-seronegative adults showed 50% lower HSV-2 acquisition rate among vaccine vs placebo in initial 5 months. Overall vaccine efficacy 9% and no influence on duration of first HSV-2 episode or frequency of reactivation | (2, 3) |
| HCMV | gB | Sanofi / Chiron.  Subunit vaccine administered with MF-59 adjuvant | Phase II | Phase II Clinical trial in HCMV-seronegative women less than 1 year after child birth showed efficacy of 50% in 42-month follow up. Same vaccine formulation and 3-dose regimen tested in adolescent girls aged 14-17 showed 43% efficacy after 2 years follow up post-third dose. Phase II study of 3-dose regimen in transplant recipients showed anti-gB titres higher in vaccinated cohort compared to placebo. Viremia duration inversely correlated with anti-gB titre and number of required days of ganciclovir treatment significantly reduced in vaccinated cohort compared with placebo. Separate study investigating immune response to vaccine 0-90 days after solid-organ transplant showed faster induction of anti-gB humoral response compared to placebo with proportion of vaccinees raising neutralising anti-gB response. | (4-7)  NCT00125502 NCT00133497 NCT00815165 NCT00299260 |
| HCMV | gB and phosphoprotein 65 | Vical.  DNA Vaccine with CRL1005 Poloxamer formulation and benzalkonium chloride surfactant to induce nanoparticle formation | Phase II & III | Phase II Trial Investigated 3-dose regimen in HCMV-positive hematopoietic cell transplant (HCT) recipients aged 18-56 with lymphoma or leukaemia, administered 1, 3 and 6 months post-transplant. Rate of HCMV-specific initiation of antiviral therapy lower in vaccinated group but non-significantly (p=0.145). Significant reduction to occurrence of HCMV viremia as measured by PCR amplification of HCMV DNA from patient plasma. Significant reduction to duration of viremia and increase to interval between viraemic episodes. Phase III trial in seropositive HCT transplant recipients with 5-dose regimen showed no significant reduction to composite all-cause mortality and adjudicated HCMV end-organ disease primary endpoint compared with placebo, 1 year post-transplant. pp-65 T-Contradictory to Phase II study, pp-65-specific T Cell response higher in placebo group compared to intervention group. Failure to protect attributed to the poor induction of anti-gB antibodies, non-significant when compared to placebo. IFN-gamma-producing T Cells specific to pp65 correlated with protection. In follow-up clinical trial in seronegative kidney transplant recipients receiving HCMV-positive organs, no significant difference in primary endpoint was observed between vaccinated and placebo cohort in a >1 year follow-up. Also attributed to poor induction of a humoral response | (8-13)  NCT00285259 NCT02103426 NCT01903928 NCT01974206 NCT01877655 |
| HCMV | gB chimeric protein fused to HSV gD | GSK.  AS01-E adjuvanted subunit vaccine. | Phase I non-randomised, open label | No adverse reactions in study of 3 15ug-dose regimen. Follow-up of a subset of vaccinated participants for 60 months showed elevated anti-gB IgG responses by ELISA compared to naturally infected individuals identified at the initial study screen, however differences were not significant. | NCT00435396 NCT01357915 GSK Study ID: 108890 |
| HCMV | gB, pentameric complex recombinant proteins | GSK.  Adjuvanted subunit vaccine. | Phase I/II | Safety study comparing different doses of gB and pentameric complex in combination. All study arms are administered vaccine at 0, 2 and 6 months, with a minimum follow up to month 18. | NCT05089630 (completed 2025, results expected 2026). |
| HCMV | gB or phosphoprotein 65/IE1 fusion protein | Alphavax.  Alphavirus replicon particle vaccine | Phase I | Seronegative adult participants randomised to vaccine or placebo cohort 4:1 in observer-blinded trial. All participants in vaccine group showed HCMV-specific IFN-gamma production upon T cell stimulation as well as neutralising antibody induction. | (14)  NCT00439803 |
| HCMV | gB and phosphoprotein 65 | Hookipa.  Two non-replicating lymphocytic choriomeningitis virus vectors | Phase I Phase II Terminated | Three doses administered on a 0, 1, 3-month dose regimen to healthy adults. Dose-dependent cell mediated immune response is dominated by pp-65-specific CD8 T cell response. Neutralising anti-gB antibodies induced after two doses, induced in dose-dependent manner. 1 of the 42 participants generated anti-LCMV vector response.  Phase II trial on seronegative kidney transplant patients receiving seropositive organs showed no reduction to HCMV viremia, HCMV disease or use of antivirals after two vaccine doses. Reduction in viremia after three doses, however vaccine development halted. | (15, 16)  NCT03629080  https://www.globenewswire.com/news-release/2023/03/15/2627479/0/en/HOOKIPA-Reports-Fourth-Quarter-and-Full-Year-2022-Financial-Results-and-Provides-2023-Outlook.html |
| HCMV | gB ectodomain fused to VSV-G transmembrane and C-terminal domain | VBI.  eVLP derived from murine leukaemia virus gag protein adjuvanted with alum | Phase I | Three-shot regimen tested in healthy adults with three different doses of gB- VSV G fusion protein. High dose regimen (2ug) elicited neutralising Abs against fibroblast infection in 100% of patients and against epithelial cell infection in 31% of patients.  Exploratory immunology on samples from Phase I study comparing eVLP vaccine to gB/MF59 shows that IgG titres against gB ectodomain and full-length gB are lower than those induced by gB/MF59, as was ADCP activity. Vaccination did not induce ADCC responses in participants, and vaccination did not induce IgG against AD2 (known neutralising site) or AD6 (associated with disrupting cell-to-cell spread). Titres to cell-expressed gB, AD4+5 were higher than those raised against gB/MF59. | Phase I: NCT00133497  (17, 18) |
| HCMV | gB, pentameric complex, pp65 | Moderna.  Modified nucleoside mRNA vaccine encoding these antigens in various combinations encapsulated in lipid nanoparticle (LNP) | Phase I - III | mRNA-1647 vaccine induces both anti-gB and anti-PC antibody responses in seropositive and seronegative participants, over the 18-month follow-up period. Induction of both neutralising anti-gB antibody and antibody capable of ADCC, however lower overall anti-gB IgG responses and lower ADCP activity compared to gB/MF59. Extended follow-up on a subset of participants in Phase II study show stable geometric mean titres between month 18 and month 36.  Phase III study enrolled 7454 women aged 16-40 expected to read out in 2028. Efficacy endpoint is seroconversion against non-vaccine HCMV antigens. Separate Phase II study with efficacy endpoints in hematopoietic stem cell transplant patients, expected to read out in 2026. | Phase I: NCT03382405, (19, 20) NCT05105048 and NCT05575492  Phase II: NCT04232280, NCT04975893 (Extension study, data presented at ESCMID conference 2025).  Phase III & Phase II with efficacy endpoint: NCT05085366, NCT05683457 |

**Supplementary References**

1. Mertz GJ, Ashley R, Burke RL, Benedetti J, Critchlow C, Jones CC, et al. Double-Blind, Placebo-Controlled Trial of a Herpes Simplex Virus Type 2 Glycoprotein Vaccine in Persons at High Risk for Genital Herpes Infection. Journal of Infectious Diseases. 1990;161(4):653–60.

2. Corey L, Langenberg AGM, Ashley R, Sekulovich RE, Izu AE, Douglas JM, et al. Recombinant glycoprotein vaccine for the prevention of genital HSV-2 infection: Two randomized controlled trials. Journal of the American Medical Association. 1999;282(4):331–40.

3. Straus SE, Wald A, Kost RG, McKenzie R, Langenberg AGM, Hohman P, et al. Immunotherapy of recurrent genital herpes with recombinant herpes simplex virus type 2 glycoproteins D and B: Results of a placebo-controlled vaccine trial. Journal of Infectious Diseases. 1997;176(5):1129–34.

4. Baraniak I, Gomes AC, Sodi I, Langstone T, Rothwell E, Atkinson C, et al. Seronegative patients vaccinated with cytomegalovirus gB-MF59 vaccine have evidence of neutralising antibody responses against gB early post-transplantation. EBioMedicine. 2019;50:45–54.

5. Griffiths PD, Stanton A, McCarrell E, Smith C, Osman M, Harber M, et al. Cytomegalovirus glycoprotein-B vaccine with MF59 adjuvant in transplant recipients: A phase 2 randomised placebo-controlled trial. The Lancet. 2011;377(9773):1256–63.

6. Pass RF, Zhang C, Evans A, Simpson T, Andrews W, Huang M-L, et al. Vaccine Prevention of Maternal Cytomegalovirus Infection. New England Journal of Medicine. 2009;360(12):1191–9.

7. Cardin RD, Bravo FJ, Pullum DA, Orlinger K, Watson EM, Aspoeck A, et al. Replication-defective lymphocytic choriomeningitis virus vectors expressing guinea pig cytomegalovirus gB and pp65 homologs are protective against congenital guinea pig cytomegalovirus infection. Vaccine. 2016;34(17):1993–9.

8. Kharfan-Dabaja MA, Boeckh M, Wilck MB, Langston AA, Chu AH, Wloch MK, et al. A novel therapeutic cytomegalovirus DNA vaccine in allogeneic haemopoietic stem-cell transplantation: A randomised, double-blind, placebo-controlled, phase 2 trial. The Lancet Infectious Diseases. 2012;12(4):290–9.

9. Ljungman P, Bermudez A, Logan AC, Kharfan-Dabaja MA, Chevallier P, Martino R, et al. A randomised, placebo-controlled phase 3 study to evaluate the efficacy and safety of ASP0113, a DNA-based CMV vaccine, in seropositive allogeneic haematopoietic cell transplant recipients. EClinicalMedicine. 2021;33:100787–.

10. Plotkin SA, Wang D, Oualim A, Diamond DJ, Kotton CN, Mossman S, et al. The Status of Vaccine Development Against the Human Cytomegalovirus. The Journal of Infectious Diseases. 2020;221(Supplement_1):S113–S22.

11. Smith L, Wloch M, Chaplin J, Gerber M, Rolland A. Clinical Development of a Cytomegalovirus DNA Vaccine: From Product Concept to Pivotal Phase 3 Trial. Vaccines. 2013;1(4):398–414.

12. Vincenti F, Budde K, Merville P, Shihab F, Ram Peddi V, Shah M, et al. A randomized, phase 2 study of ASP0113, a DNA-based vaccine, for the prevention of CMV in CMV-seronegative kidney transplant recipients receiving a kidney from a CMV-seropositive donor. American Journal of Transplantation. 2018;18(12):2945–54.

13. Wloch MK, Smith LR, Boutsaboualoy S, Reyes L, Han C, Kehler J, et al. Safety and immunogenicity of a bivalent cytomegalovirus DNA vaccine in healthy adult subjects. Journal of Infectious Diseases. 2008;197(12):1634–42.

14. Bernstein DI, Reap EA, Katen K, Watson A, Smith K, Norberg P, et al. Randomized, double-blind, Phase 1 trial of an alphavirus replicon vaccine for cytomegalovirus in CMV seronegative adult volunteers. Vaccine. 2009;28(2):484–93.

15. Schwendinger M, Thiry G, De Vos B, Leroux-Roels G, Bruhwyler J, Huygens A, et al. A Randomized Dose-Escalating Phase I Trial of a Replication-Deficient Lymphocytic Choriomeningitis Virus Vector-Based Vaccine Against Human Cytomegalovirus. The Journal of Infectious Diseases. 2020;XX:1–12.

16. Kotton CN, Kumar D, Caliendo AM, Huprikar S, Chou S, Danziger-Isakov L, et al. The Third International Consensus Guidelines on the Management of Cytomegalovirus in Solid-organ Transplantation. Transplantation. 2018;102(6):900–31.

17. Connors MR, Karthigeyan KP, Fuller AS, Mitchell L, Preston H, Ananyev S, et al. Specificity and functional humoral immune responses induced by the VBI-1501A eVLP HCMV gB vaccine compared to the gB/MF59 vaccine. Human Vaccines & Immunotherapeutics. 2025;21(1).

18. Langley JM, Gantt S, Halperin SA, Ward B, McNeil S, Ye L, et al. An enveloped virus-like particle alum-adjuvanted cytomegalovirus vaccine is safe and immunogenic: A first-in-humans Canadian Immunization Research Network (CIRN) study. Vaccine. 2024;42(3):713–22.

19. Hu X, Karthigeyan KP, Herbek S, Valencia SM, Jenks JA, Webster H, et al. Human Cytomegalovirus mRNA-1647 Vaccine Candidate Elicits Potent and Broad Neutralization and Higher Antibody-Dependent Cellular Cytotoxicity Responses Than the gB/MF59 Vaccine. The Journal of Infectious Diseases. 2024;230(2):455–66.

20. Fierro C, Brune D, Shaw M, Schwartz H, Knightly C, Lin J, et al. Safety and Immunogenicity of a Messenger RNA–Based Cytomegalovirus Vaccine in Healthy Adults: Results From a Phase 1 Randomized Clinical Trial. The Journal of Infectious Diseases. 2024;230(3):e668–e78.
