# Supplementary material for: Progress and prospects for herpesvirus vaccination using gB antigens
Source: Front Immunol. 2026 May 29;17:1827628. doi: 10.3389/fimmu.2026.1827628 (PMC13260650; doi:10.3389/fimmu.2026.1827628)
Supplement: Supplementary Table 2 — Relating to Section 4, detailed characterisation and comparison of immune responses elicited in HCMV gB trials. [file Table2.docx]

**Supplementary Table 2 - Detailed characterisation and comparison of immune responses elicited in HCMV gB trials.** Vaccine responses described are those in participants seronegative at baseline. Green highlights notable positive findings or favourable comparisons; blue indicates negative or unfavourable findings.

Unless otherwise stated, gB/MF59 responses were from the study performed in seronegative postpartum women, as characterised by Nelson et al (1, 2). For unclear reasons, the pattern of responses was markedly different in the study performed in adolescent women (3, 4), with most responses stronger in the adolescent cohort (exceptions which were stronger in the postpartum cohort were TB40/E strain ADCP, AD1 and AD4 binding). Comparisons to seropositive reference samples have not, to our knowledge, been described for the post-partum cohort. Given the immunological complexity of the context of solid-organ transport, we have not attempted to summarize gB/MF59 responses seen in that population, except for AD6 binding and inhibition of cell-to-cell spread (which we are not aware of having been measured in the other trials’ populations).

VBI1501A responses were after the third dose of 2.0 µg, formulated with alum, i.e. the highest responses seen in the first-in-human trial (NCT 02826798) (5).

mRNA-1647 responses are those one month after the third dose of 180 µg in study NCT03382405 (6, 7).

‘Seropositive’ responses, where referred to, are those in an unvaccinated seropositive comparator sample set (not standardised between studies).

gB/MF59 responses used as comparators for mRNA-1647 responses by Hu et al and for VBI1501A responses by Connors et al were taken one month after the third dose of 20 µg in the gB/MF59 trial in adolescents (NCT00133497) (4, 8).

*’Post-fusion protein’ refers to the Sanofi gB/MF59 antigen (which comprised the ectodomain and intravirion domain), the recombinant protein sold by SinoBiological, which is similar to the Sanofi antigen (catalogue number 10202-V08H1, comprising amino acids 1-700 and 777-907), or a post-fusion ectodomain-only construct similar to that produced by Chandramouli et al (9).

|  |  | **Vaccine (immunogen strain), with reference to primary publication providing immunological data** | | |
| --- | --- | --- | --- | --- |
| **Assay group** | **Assay** | **gB/MF59 (Towne)** | **mRNA-1647 (Merlin)** | **VBI1501A (Towne)** |
| IgG binding | Post-fusion protein* | Similar to seropositive in post-partum cohort (Nelson), higher than seropositive in adolescent cohort (Hu). | Similar to seropositive, c. 10-fold weaker than gB/MF59 | Post-fusion ectodomain trimer positive, weaker than gB/MF59 (Connors). SinoBiological full-length positive, weaker than gB/MF59 (Connors) |
| IgG binding | Pre-fusion stabilised ectodomain | To our knowledge, not done. | To our knowledge, not done. | Positive, no comparator (Connors) |
| IgG binding | gB-transfected cells | Positive but weaker than seropositive in postpartum vaccinees (Nelson). Stronger than seropositive in adolescent vaccinees (Hu). | On Towne, similar to seropositive but weaker than gB/MF59. On Merlin, similar pattern but smaller differences. | 97% positive vs Towne, stronger than gB/MF59  57% positive vs Merlin, stronger than gB/MF59 (Connors) |
| IgG binding | Virion-binding ELISA | Weaker than seropositive (Hu) | Weaker than seropositive or gB/MF59 | Below cutoff (Connors) |
| IgG binding | AD1 (DIV) | Some positive, similar to seropositive in adolescents (Hu), weaker than seropositive postpartum (Nelson) | Some positive, similar to gB/MF59 (Hu) | Undetectable (Connors) |
| IgG binding | AD2 (NTD) | Minority positive for AD2S1 (Hu)  AD2 weaker than seropositive in postpartum (Nelson) | Small minority positive for AD2S1 | For AD2 (not S1-spec), weaker than seropos (Langley)  For AD2S1, undetectable (Connors) |
| IgG binding | AD4 (DII) | Positive (Hu).  Similar to seropositive in postpartum vaccinees (Nelson). | Positive, similar to gB/MF59 (Hu) | Positive, higher than gB/MF59 (Connors) |
| IgG binding | AD5 (DI) | Positive (Hu).  DI positive but weaker than seropositive in postpartum vaccinees (Nelson). | Weaker than gB/MF59 (Hu) | Positive (Connors) |
| IgG binding | AD4+5 | Positive (Hu) | Mixed, slightly weaker than gB/MF59 | Positive, higher than gB/MF59 (Connors) |
| IgG binding | AD6 (DV) | Positive (Hu) Stronger than seropositives (Gomes) | Weaker than gB/MF59 (Hu) | Undetectable (Connors) |
|  |  |  |  |  |
| Functional | Neut, fibroblast | Weak / many negative, weaker than seropositive (AD169r and Towne [Hu & Nelson]) | Positive ‘low level’ (log_10_ NT50 c. 2-3) against Towne and AD169r : 400-fold lower than on epithelial, similar to seropos, greater than gB/MF59 (Note pentamer response present) | Sl. higher than seropos (Langley) |
| Functional | Neut, epithelial | Weak / many negative, weaker than seropositive (AD169r, Hu and TB40/E, Nelson) | Positive, ‘potent’ (NT50 c. 4-5), greater than seropos and gB/MF59 vs AD169r. (Note pentamer response present) | Present in minority (Langley) |
| Functional | ADCC  (PBMC & Merlin-infected HFFF co-culture, CD56^+^ CD107^+^ flow) | Very weak, well below seropositive (c. 1.5% vs 20%) (Hu) | Positive (note pentamer response present). More strongly correlated with anti-gB (particularly FcγR3a-binding) than anti-PC IgG titres. Weaker than seropositive, slightly stronger than gB/MF59 | Negative (Merlin, seropos samples positive)(Connors) |
| Functional | ADCP (Fluorescently-labelled AD169r virions with THP-1 cells) | Weaker than seropositive (Hu) | Positive (note pentamer response present). More strongly correlated with anti-gB (particularly and FcγR2a-binding) than anti-PC IgG titres. Weaker than seropositive, slightly weaker than gB/MF59. | Positive (Connors) |
| Functional | Cell-to-cell spread inhibition | Some evidence of effect in selected samples in SOT population at peak timepoint (Gomes). |  | No effect on Merlin disseminating exclusively cell-cell (Connors) |

**Supplementary References**

1. Nelson CS, Herold BC, Permar SR. A new era in cytomegalovirus vaccinology: considerations for rational design of next-generation vaccines to prevent congenital cytomegalovirus infection. npj Vaccines: Nature Publishing Group; 2018. p. 38–.

2. Pass RF, Zhang C, Evans A, Simpson T, Andrews W, Huang M-L, et al. Vaccine Prevention of Maternal Cytomegalovirus Infection. New England Journal of Medicine. 2009;360(12):1191–9.

3. Jenks JA, Nelson CS, Roark HK, Goodwin ML, Pass RF, Bernstein DI, et al. Antibody binding to native cytomegalovirus glycoprotein B predicts efficacy of the gB/MF59 vaccine in humans. Science Translational Medicine. 2020;12(568):eabb3611–eabb.

4. Bernstein DI, Munoz FM, Callahan ST, Rupp R, Wootton SH, Edwards KM, et al. Safety and efficacy of a cytomegalovirus glycoprotein B (gB) vaccine in adolescent girls: A randomized clinical trial. Vaccine. 2016;34(3):313–9.

5. Langley JM, Gantt S, Halperin SA, Ward B, McNeil S, Ye L, et al. An enveloped virus-like particle alum-adjuvanted cytomegalovirus vaccine is safe and immunogenic: A first-in-humans Canadian Immunization Research Network (CIRN) study. Vaccine. 2024;42(3):713–22.

6. Fierro C, Brune D, Shaw M, Schwartz H, Knightly C, Lin J, et al. Safety and Immunogenicity of a Messenger RNA–Based Cytomegalovirus Vaccine in Healthy Adults: Results From a Phase 1 Randomized Clinical Trial. The Journal of Infectious Diseases. 2024;230(3):e668–e78.

7. Hu X, Karthigeyan KP, Herbek S, Valencia SM, Jenks JA, Webster H, et al. Human Cytomegalovirus mRNA-1647 Vaccine Candidate Elicits Potent and Broad Neutralization and Higher Antibody-Dependent Cellular Cytotoxicity Responses Than the gB/MF59 Vaccine. The Journal of Infectious Diseases. 2024;230(2):455–66.

8. Connors MR, Karthigeyan KP, Fuller AS, Mitchell L, Preston H, Ananyev S, et al. Specificity and functional humoral immune responses induced by the VBI-1501A eVLP HCMV gB vaccine compared to the gB/MF59 vaccine. Human Vaccines & Immunotherapeutics. 2025;21(1).

9. Chandramouli S, Ciferri C, Nikitin PA, Caló S, Gerrein R, Balabanis K, et al. Structure of HCMV glycoprotein B in the postfusion conformation bound to a neutralizing human antibody. Nature Communications. 2015;6(1):1–12.
